# Bis(μ-2-carb­oxy­methyl-2-hy­droxy­butane­dioato)bis­[diaqua­manganese(II)]–1,2-bis­(pyridin-4-yl)ethene–water (1/1/2)

**DOI:** 10.1107/S1600536812047034

**Published:** 2012-11-24

**Authors:** In Hong Hwang, Pan-Gi Kim, Jae-Cheon Lee, Cheal Kim, Youngmee Kim

**Affiliations:** a Department of Fine Chemistry, Seoul National University of Science & Technology, Seoul 139-743, Republic of Korea; b Department of Forest & Environment Resources, Kyungpook National University, Sangju 742-711, Republic of Korea; c Department of Forest Resources Development, Korea Forest Research Institute, Suwon 441-350, Republic of Korea; d Department of Chemistry and Nano Science, Ewha Womans University, Seoul 120-750, Republic of Korea

## Abstract

The asymmetric unit of the title compound, [Mn_2_(C_6_H_6_O_7_)_2_(H_2_O)_4_]·C_12_H_10_N_2_·2H_2_O, contains half of the centrosymmetric Mn complex dimer, half of a 1,2-bis­(pyridin-4-yl)ethene mol­ecule, which lies across an inversion center, and one water mol­ecule. Two citrate ligands bridge two Mn^II^ ions, and each Mn^II^ atom is coordinated by four O atoms from the citrate ligands (one from hy­droxy and three from carboxyl­ate groups) and two water O atoms, forming a distorted octa­hedral environment. In the crystal, O—H⋯O and O—H⋯N hydrogen bonds link the centrosymmetric dimers and lattice water mol­ecules into a three-dimensional structure which is further stabilized by inter­molecular π–π inter­actions [centroid–centroid distance = 3.959 (2) Å]. Weak C—H⋯O hydrogen bonding interactions are also observed.

## Related literature

For inter­actions of metal ions with biologically active mol­ecules, see: Daniele *et al.* (2008 ▶); Parkin (2004 ▶); Tshuva & Lippard (2004 ▶); Stoumpos *et al.* (2009 ▶). For manganese citrate and zinc citrate complexes, see: Hwang *et al.* (2012*a* ▶,*b* ▶). For related complexes, see: Yu *et al.* (2009 ▶); Kim *et al.* (2011 ▶).
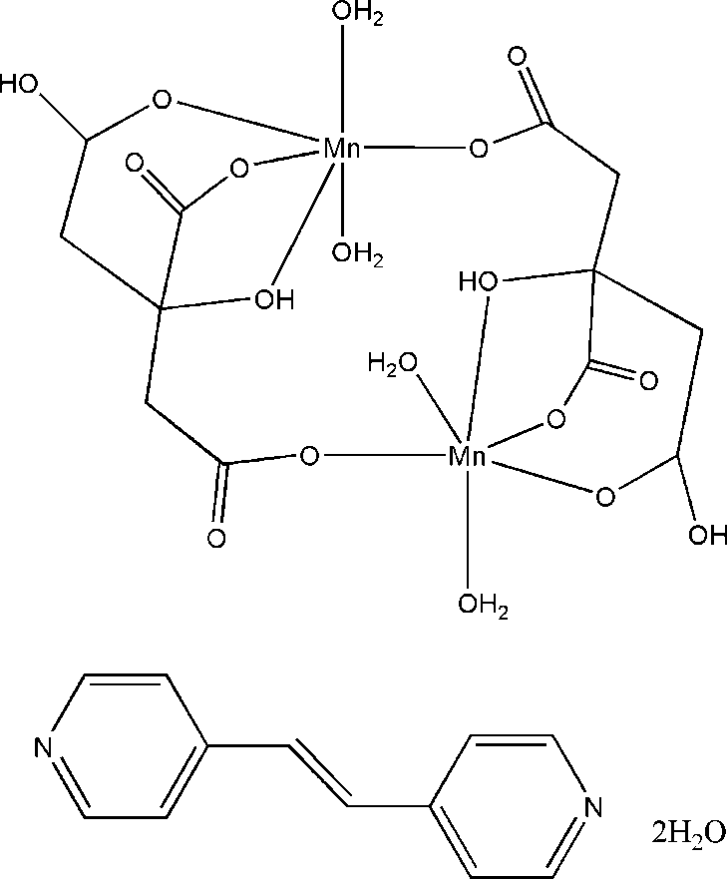


## Experimental

### 

#### Crystal data

[Mn_2_(C_6_H_6_O_7_)_2_(H_2_O)_4_]·C_12_H_10_N_2_·2H_2_O
*M* *_r_* = 780.41
Triclinic, 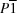

*a* = 9.3970 (19) Å
*b* = 9.4580 (19) Å
*c* = 10.131 (2) Å
α = 70.24 (3)°
β = 67.11 (3)°
γ = 75.52 (3)°
*V* = 773.3 (3) Å^3^
*Z* = 1
Mo *K*α radiation
μ = 0.91 mm^−1^
*T* = 170 K
0.15 × 0.10 × 0.02 mm

#### Data collection

Bruker SMART CCD diffractometer
Absorption correction: multi-scan (*SADABS*; Bruker, 1997 ▶) *T*_min_ = 0.876, *T*_max_ = 0.982
4358 measured reflections
2973 independent reflections
2423 reflections with *I* > 2σ(*I*)
*R*_int_ = 0.021

#### Refinement

*R*[*F* ^2^ > 2σ(*F* ^2^)] = 0.038
*wR*(*F* ^2^) = 0.098
*S* = 1.08
2973 reflections
241 parameters
8 restraints
H atoms treated by a mixture of independent and constrained refinement
Δρ_max_ = 0.47 e Å^−3^
Δρ_min_ = −0.63 e Å^−3^

### 

Data collection: *SMART* (Bruker, 1997 ▶); cell refinement: *SAINT* (Bruker, 1997 ▶); data reduction: *SAINT*; program(s) used to solve structure: *SHELXS97* (Sheldrick, 2008 ▶); program(s) used to refine structure: *SHELXL97* (Sheldrick, 2008 ▶); molecular graphics: *SHELXTL* (Sheldrick, 2008 ▶); software used to prepare material for publication: *SHELXTL*.

## Supplementary Material

Crystal structure: contains datablock(s) I, global. DOI: 10.1107/S1600536812047034/sj5281sup1.cif

Structure factors: contains datablock(s) I. DOI: 10.1107/S1600536812047034/sj5281Isup2.hkl

Additional supplementary materials:  crystallographic information; 3D view; checkCIF report

## Figures and Tables

**Table 1:** Hydrogen-bond geometry (Å, °)

*D*—H⋯*A*	*D*—H	H⋯*A*	*D*⋯*A*	*D*—H⋯*A*
C12—H12⋯O3^i^	0.95	2.48	3.344 (4)	151
C11—H11⋯O4^ii^	0.95	2.53	3.143 (3)	123
O9—H9*B*⋯O3^iii^	0.93 (1)	2.56 (3)	3.113 (3)	118 (2)
O9—H9*B*⋯O2^iii^	0.93 (1)	1.88 (1)	2.803 (3)	175 (3)
O9—H9*A*⋯O1*W* ^iv^	0.93 (1)	1.78 (1)	2.695 (3)	170 (3)
O8—H8*B*⋯O5^v^	0.93 (1)	1.78 (1)	2.707 (3)	173 (3)
O8—H8*A*⋯O7^iv^	0.93 (1)	1.87 (1)	2.769 (3)	163 (3)
O5—H5*O*⋯N11^vi^	0.86 (1)	1.76 (1)	2.625 (3)	178 (3)
O1*W*—H1*WB*⋯O3	0.93 (1)	1.92 (1)	2.843 (3)	175 (3)
O1—H1*O*⋯O6	0.86 (1)	1.88 (2)	2.616 (2)	143 (2)
O1*W*—H1*WA*⋯O7^vii^	0.93 (1)	2.08 (2)	2.891 (3)	145 (3)

Symmetry codes: (i) 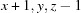; (ii) 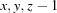; (iii) 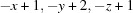; (iv) 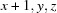; (v) 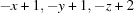; (vi) 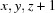; (vii) 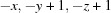.

**Fig. 1.**
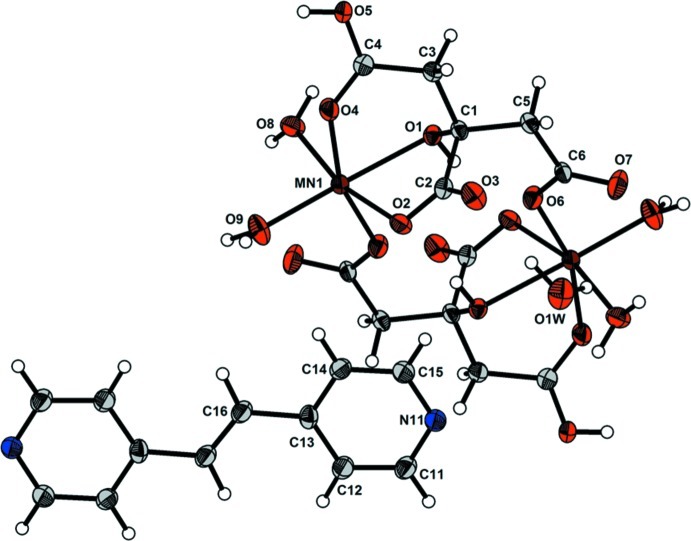
The structure of the title compound showing the atom numbering scheme. Displacement ellipsoids are shown at the 50% probability level. The labelled atoms are related to unlabelled atoms by the symmetry codes: [1 - x, 1 - y, 1 - z] for diaqua-bis-(citrato)di-manganese(II) complex and [2 - x, 2 - y,z] for the 1,2-bis(pyridin-4-yl)ethene molecule.

## supplementary crystallographic information

### Comment

Complexes involving citric acid have often been used as models to examine the interaction between transition metal ions with biologically active molecules (Daniele *et al.*, 2008; Parkin, 2004; Tshuva & Lippard, 2004; Stoumpos *et al.*, 2009). Quite recently, our group has reported two novel compounds from the reaction of manganese(II) and zinc(II) nitrates as the building blocks and citric acid as the ligand (Hwang, *et al.*, 2012*a*,*b*). In order to study the effects of spacer ligands on the interaction between transition metal ions with citric acid (Yu, *et al.*, 2009; Kim, *et al.*, 2011), in this work, we have attempted to employ 1,2-bis(4-pyridyl)ethene as a spacer source. We report here the resulting structure in which the 1,2-bis(4-pyridyl)ethene molecule does not function as a spacer but co-crystallises with the Mn complex to form bis(µ-2-carboxymethyl-2-hydroxybutanedioato)bis[diaquamanganese(II)]– 1,2-bis(pyridin-4-yl)ethene–water(1/1/2).

The molecular structure of the title compound is shown in Fig. 1. The asymmetric unit of the title compound, C_24_H_34_Mn_2_N_2_O_20_, contains half of the centrosymmetric Mn complex dimer, half of a 1,2-bis(pyridin-4-yl)ethene molecule, which lies across an inversion center, and one water molecule. Two citrate ligands bridge two Mn^II^ ions, and each Mn^II^ is coordinated by four oxygen atoms from the citrate ligands (one hydroxyl and three carboxylate, with one bridging) and two water oxygen atoms, forming a distorted octahedral environment. In the crystal, O—H···O hydrogen bonds link the centrosymmetric dimers and lattice water molecules into a three-dimensional structure. The crystal structure is further stabilized by intermolecular *π*-*π* interactions [centroid = C11–C15/N11; centroid–centroid distance = 3.959 (2) Å symmetry code: 1 - *x*, 2 - *y*, *z*].

### Experimental

Citric acid (19.4 mg, 0.1 mmol) and Zn(NO_3_)_2_.6H_2_O (30.4 mg, 0.1 mmol) were dissolved in 4 ml H_2_O and carefully layered by 4 ml of an acetonitrile solution of 1,2-bis(4-pyridyl)ethene (37.6 mg, 0.2 mmol). Suitable crystals of the title compound were obtained from this solution within two weeks.

### Refinement

H atoms bound to C were placed in calculated positions with C—H distances of 0.95 Å for aromatic C atoms and 0.99 Å for methylene C atoms. They were included in the refinement using the riding-motion approximation with *U*iso(H) = 1.2*U*eq(C). H atoms bound to O were located in difference Fourier maps and refined with their O–H distances restrained as follows and *U*iso(H) = 1.2*U*eq(O). Hydroxyl O—H = 0.860 (2) Å, coordinated water molecules O—H = 0.930 (2) Å the free water molecule O—H = 0.930 (2) Å.

### Crystal data

**Table:**

[Mn_2_(C_6_H_6_O_7_)_2_(H_2_O)_4_]·C_12_H_10_N_2_·2H_2_O	*Z* = 1
*M**_r_* = 780.41	*F*(000) = 402
Triclinic, *P*1	*D*_x_ = 1.676 Mg m^−^^3^
Hall symbol: -P 1	Mo *K*α radiation, λ = 0.71073 Å
*a* = 9.3970 (19) Å	Cell parameters from 11909 reflections
*b* = 9.4580 (19) Å	θ = 2.7–27.6°
*c* = 10.131 (2) Å	µ = 0.91 mm^−^^1^
α = 70.24 (3)°	*T* = 170 K
β = 67.11 (3)°	Plate, colorless
γ = 75.52 (3)°	0.15 × 0.10 × 0.02 mm
*V* = 773.3 (3) Å^3^	

### Data collection

**Table:**

Bruker SMART CCD diffractometer	2973 independent reflections
Radiation source: fine-focus sealed tube	2423 reflections with *I* > 2σ(*I*)
Graphite monochromator	*R*_int_ = 0.021
φ and ω scans	θ_max_ = 26.0°, θ_min_ = 2.3°
Absorption correction: multi-scan (*SADABS*; Bruker, 1997)	*h* = −11→10
*T*_min_ = 0.876, *T*_max_ = 0.982	*k* = −11→10
4358 measured reflections	*l* = −12→12

### Refinement

**Table:**

Refinement on *F*^2^	Primary atom site location: structure-invariant direct methods
Least-squares matrix: full	Secondary atom site location: difference Fourier map
*R*[*F*^2^ > 2σ(*F*^2^)] = 0.038	Hydrogen site location: inferred from neighbouring sites
*wR*(*F*^2^) = 0.098	H atoms treated by a mixture of independent and constrained refinement
*S* = 1.08	*w* = 1/[σ^2^(*F*_o_^2^) + (0.0538*P*)^2^] where *P* = (*F*_o_^2^ + 2*F*_c_^2^)/3
2973 reflections	(Δ/σ)_max_ = 0.001
241 parameters	Δρ_max_ = 0.47 e Å^−^^3^
8 restraints	Δρ_min_ = −0.63 e Å^−^^3^

### Special details

**Table:**

Geometry. All e.s.d.'s (except the e.s.d. in the dihedral angle between two l.s. planes) are estimated using the full covariance matrix. The cell e.s.d.'s are taken into account individually in the estimation of e.s.d.'s in distances, angles and torsion angles; correlations between e.s.d.'s in cell parameters are only used when they are defined by crystal symmetry. An approximate (isotropic) treatment of cell e.s.d.'s is used for estimating e.s.d.'s involving l.s. planes.
Refinement. Refinement of *F*^2^ against ALL reflections. The weighted *R*-factor *wR* and goodness of fit *S* are based on *F*^2^, conventional *R*-factors *R* are based on *F*, with *F* set to zero for negative *F*^2^. The threshold expression of *F*^2^ > σ(*F*^2^) is used only for calculating *R*-factors(gt) *etc*. and is not relevant to the choice of reflections for refinement. *R*-factors based on *F*^2^ are statistically about twice as large as those based on *F*, and *R*- factors based on ALL data will be even larger.

### Fractional atomic coordinates and isotropic or equivalent isotropic displacement parameters (Å^2^)

**Table:**

	*x*	*y*	*z*	*U*_iso_*/*U*_eq_	
Mn1	0.59596 (4)	0.68914 (4)	0.60932 (4)	0.01775 (14)	
O1	0.4231 (2)	0.51640 (19)	0.72246 (19)	0.0179 (4)	
H1O	0.435 (3)	0.479 (3)	0.652 (2)	0.021*	
O2	0.3899 (2)	0.79755 (19)	0.54416 (19)	0.0226 (4)	
O3	0.1299 (2)	0.8203 (2)	0.6391 (2)	0.0306 (5)	
O4	0.4605 (2)	0.7549 (2)	0.81346 (19)	0.0248 (4)	
O5	0.3362 (2)	0.6926 (2)	1.0561 (2)	0.0289 (5)	
H5O	0.411 (2)	0.731 (3)	1.055 (3)	0.035*	
O6	0.3225 (2)	0.4206 (2)	0.56485 (19)	0.0232 (4)	
O7	0.0686 (2)	0.4237 (2)	0.6196 (2)	0.0335 (5)	
O8	0.7614 (2)	0.5543 (2)	0.7192 (2)	0.0263 (4)	
H8A	0.8656 (9)	0.526 (3)	0.669 (3)	0.032*	
H8B	0.736 (3)	0.4655 (17)	0.794 (2)	0.032*	
O9	0.7071 (2)	0.8889 (2)	0.5139 (2)	0.0311 (5)	
H9A	0.8153 (4)	0.881 (3)	0.478 (3)	0.037*	
H9B	0.671 (3)	0.9920 (8)	0.501 (3)	0.037*	
C1	0.2687 (3)	0.6001 (3)	0.7591 (3)	0.0166 (5)	
C2	0.2613 (3)	0.7522 (3)	0.6372 (3)	0.0187 (5)	
C3	0.2331 (3)	0.6280 (3)	0.9100 (3)	0.0209 (6)	
H3A	0.1328	0.6957	0.9320	0.025*	
H3B	0.2183	0.5299	0.9871	0.025*	
C4	0.3533 (3)	0.6963 (3)	0.9249 (3)	0.0195 (5)	
C5	0.1477 (3)	0.5074 (3)	0.7752 (3)	0.0184 (5)	
H5A	0.1411	0.4199	0.8642	0.022*	
H5B	0.0447	0.5713	0.7934	0.022*	
C6	0.1791 (3)	0.4481 (3)	0.6430 (3)	0.0188 (5)	
N11	0.5629 (3)	0.8142 (2)	0.0477 (2)	0.0236 (5)	
C14	0.6748 (3)	0.8996 (3)	0.1754 (3)	0.0234 (6)	
H14	0.6673	0.9197	0.2638	0.028*	
C11	0.6896 (3)	0.8400 (3)	−0.0763 (3)	0.0257 (6)	
H11	0.6931	0.8193	−0.1630	0.031*	
C12	0.8134 (3)	0.8954 (3)	−0.0799 (3)	0.0247 (6)	
H12	0.9015	0.9128	−0.1679	0.030*	
C13	0.8076 (3)	0.9261 (3)	0.0493 (3)	0.0216 (6)	
C15	0.5544 (3)	0.8440 (3)	0.1713 (3)	0.0244 (6)	
H15	0.4641	0.8267	0.2572	0.029*	
C16	0.9369 (3)	0.9793 (3)	0.0573 (3)	0.0246 (6)	
H16	0.9299	0.9845	0.1517	0.030*	
O1W	0.0209 (3)	0.8324 (2)	0.4096 (3)	0.0387 (5)	
H1WA	0.030 (4)	0.7316 (11)	0.412 (4)	0.046*	
H1WB	0.063 (4)	0.826 (4)	0.481 (3)	0.046*	

### Atomic displacement parameters (Å^2^)

**Table:**

	*U*^11^	*U*^22^	*U*^33^	*U*^12^	*U*^13^	*U*^23^
Mn1	0.0180 (2)	0.0188 (2)	0.0175 (2)	−0.00517 (15)	−0.00514 (16)	−0.00525 (16)
O1	0.0186 (9)	0.0177 (9)	0.0194 (9)	−0.0028 (7)	−0.0062 (8)	−0.0075 (7)
O2	0.0221 (10)	0.0213 (9)	0.0203 (10)	−0.0057 (8)	−0.0052 (8)	−0.0009 (7)
O3	0.0199 (10)	0.0247 (10)	0.0401 (12)	0.0003 (8)	−0.0103 (9)	−0.0022 (9)
O4	0.0250 (10)	0.0303 (10)	0.0222 (10)	−0.0116 (8)	−0.0026 (8)	−0.0114 (8)
O5	0.0301 (11)	0.0424 (12)	0.0210 (10)	−0.0210 (9)	−0.0075 (9)	−0.0063 (9)
O6	0.0178 (9)	0.0307 (10)	0.0245 (10)	−0.0041 (8)	−0.0045 (8)	−0.0140 (8)
O7	0.0228 (11)	0.0514 (13)	0.0378 (12)	−0.0080 (9)	−0.0096 (9)	−0.0246 (10)
O8	0.0207 (10)	0.0309 (11)	0.0215 (10)	−0.0038 (8)	−0.0063 (8)	−0.0008 (8)
O9	0.0248 (11)	0.0189 (10)	0.0454 (13)	−0.0073 (8)	−0.0074 (9)	−0.0057 (9)
C1	0.0137 (12)	0.0181 (12)	0.0174 (13)	−0.0044 (10)	−0.0024 (10)	−0.0057 (10)
C2	0.0184 (14)	0.0182 (13)	0.0219 (14)	−0.0024 (11)	−0.0080 (11)	−0.0071 (11)
C3	0.0206 (14)	0.0245 (14)	0.0181 (13)	−0.0066 (11)	−0.0039 (11)	−0.0067 (11)
C4	0.0196 (13)	0.0184 (13)	0.0213 (14)	−0.0009 (10)	−0.0071 (11)	−0.0073 (11)
C5	0.0151 (13)	0.0188 (13)	0.0196 (13)	−0.0048 (10)	−0.0021 (10)	−0.0056 (10)
C6	0.0175 (13)	0.0183 (13)	0.0209 (13)	−0.0072 (10)	−0.0055 (11)	−0.0033 (10)
N11	0.0260 (13)	0.0213 (11)	0.0242 (12)	−0.0056 (10)	−0.0111 (10)	−0.0022 (10)
C14	0.0304 (15)	0.0202 (13)	0.0228 (14)	−0.0049 (11)	−0.0129 (12)	−0.0040 (11)
C11	0.0301 (15)	0.0269 (15)	0.0251 (15)	−0.0054 (12)	−0.0121 (12)	−0.0086 (12)
C12	0.0240 (15)	0.0284 (15)	0.0246 (15)	−0.0045 (12)	−0.0090 (12)	−0.0088 (12)
C13	0.0226 (14)	0.0166 (13)	0.0289 (15)	−0.0038 (11)	−0.0126 (12)	−0.0048 (11)
C15	0.0266 (15)	0.0223 (14)	0.0243 (14)	−0.0057 (11)	−0.0096 (12)	−0.0033 (11)
C16	0.0301 (16)	0.0252 (14)	0.0241 (14)	−0.0057 (12)	−0.0133 (12)	−0.0071 (12)
O1W	0.0384 (13)	0.0336 (12)	0.0503 (14)	−0.0040 (10)	−0.0209 (11)	−0.0123 (11)

### Geometric parameters (Å, º)

**Table:**

Mn1—O9	2.136 (2)	C1—C2	1.554 (3)
Mn1—O6^i^	2.1373 (18)	C3—C4	1.510 (3)
Mn1—O8	2.163 (2)	C3—H3A	0.9900
Mn1—O4	2.1701 (19)	C3—H3B	0.9900
Mn1—O2	2.1864 (19)	C5—C6	1.521 (3)
Mn1—O1	2.288 (2)	C5—H5A	0.9900
O1—C1	1.440 (3)	C5—H5B	0.9900
O1—H1O	0.860 (2)	N11—C15	1.342 (3)
O2—C2	1.278 (3)	N11—C11	1.352 (4)
O3—C2	1.238 (3)	C14—C15	1.381 (4)
O4—C4	1.252 (3)	C14—C13	1.397 (4)
O5—C4	1.264 (3)	C14—H14	0.9500
O5—H5O	0.861 (2)	C11—C12	1.373 (4)
O6—C6	1.282 (3)	C11—H11	0.9500
O6—Mn1^i^	2.1372 (18)	C12—C13	1.412 (4)
O7—C6	1.239 (3)	C12—H12	0.9500
O8—H8A	0.929 (2)	C13—C16	1.465 (4)
O8—H8B	0.930 (2)	C15—H15	0.9500
O9—H9A	0.930 (2)	C16—C16^ii^	1.328 (5)
O9—H9B	0.930 (2)	C16—H16	0.9500
C1—C5	1.532 (3)	O1W—H1WA	0.930 (2)
C1—C3	1.533 (3)	O1W—H1WB	0.930 (2)
			
O9—Mn1—O6^i^	104.69 (8)	C4—C3—H3A	108.1
O9—Mn1—O8	95.79 (8)	C1—C3—H3A	108.1
O6^i^—Mn1—O8	95.74 (7)	C4—C3—H3B	108.1
O9—Mn1—O4	91.93 (8)	C1—C3—H3B	108.1
O6^i^—Mn1—O4	162.66 (7)	H3A—C3—H3B	107.3
O8—Mn1—O4	87.30 (8)	O4—C4—O5	122.9 (2)
O9—Mn1—O2	94.80 (8)	O4—C4—C3	121.5 (2)
O6^i^—Mn1—O2	89.16 (7)	O5—C4—C3	115.6 (2)
O8—Mn1—O2	166.80 (7)	C6—C5—C1	115.7 (2)
O4—Mn1—O2	84.48 (7)	C6—C5—H5A	108.4
O9—Mn1—O1	166.09 (7)	C1—C5—H5A	108.4
O6^i^—Mn1—O1	83.34 (7)	C6—C5—H5B	108.4
O8—Mn1—O1	94.66 (7)	C1—C5—H5B	108.4
O4—Mn1—O1	79.39 (7)	H5A—C5—H5B	107.4
O2—Mn1—O1	73.70 (7)	O7—C6—O6	123.8 (2)
C1—O1—Mn1	107.29 (13)	O7—C6—C5	119.6 (2)
C1—O1—H1O	107.4 (18)	O6—C6—C5	116.5 (2)
Mn1—O1—H1O	103.0 (18)	C15—N11—C11	120.3 (2)
C2—O2—Mn1	115.41 (15)	C15—C14—C13	119.9 (2)
C4—O4—Mn1	130.73 (16)	C15—C14—H14	120.1
C4—O5—H5O	109 (2)	C13—C14—H14	120.1
C6—O6—Mn1^i^	124.98 (16)	N11—C11—C12	121.7 (2)
Mn1—O8—H8A	123.5 (18)	N11—C11—H11	119.2
Mn1—O8—H8B	120.1 (18)	C12—C11—H11	119.2
H8A—O8—H8B	102 (2)	C11—C12—C13	119.0 (3)
Mn1—O9—H9A	120.1 (18)	C11—C12—H12	120.5
Mn1—O9—H9B	134.0 (19)	C13—C12—H12	120.5
H9A—O9—H9B	106 (3)	C14—C13—C12	118.2 (2)
O1—C1—C5	110.87 (18)	C14—C13—C16	119.2 (2)
O1—C1—C3	106.59 (19)	C12—C13—C16	122.6 (2)
C5—C1—C3	108.3 (2)	N11—C15—C14	121.0 (3)
O1—C1—C2	110.49 (19)	N11—C15—H15	119.5
C5—C1—C2	109.4 (2)	C14—C15—H15	119.5
C3—C1—C2	111.13 (19)	C16^ii^—C16—C13	124.9 (3)
O3—C2—O2	125.2 (2)	C16^ii^—C16—H16	117.5
O3—C2—C1	116.8 (2)	C13—C16—H16	117.5
O2—C2—C1	118.0 (2)	H1WA—O1W—H1WB	103 (3)
C4—C3—C1	116.8 (2)		
			
O9—Mn1—O1—C1	1.5 (4)	C3—C1—C2—O2	103.5 (2)
O6^i^—Mn1—O1—C1	−124.70 (14)	O1—C1—C3—C4	51.3 (3)
O8—Mn1—O1—C1	140.04 (14)	C5—C1—C3—C4	170.7 (2)
O4—Mn1—O1—C1	53.67 (14)	C2—C1—C3—C4	−69.1 (3)
O2—Mn1—O1—C1	−33.63 (13)	Mn1—O4—C4—O5	144.1 (2)
O9—Mn1—O2—C2	−144.09 (17)	Mn1—O4—C4—C3	−37.3 (3)
O6^i^—Mn1—O2—C2	111.24 (17)	C1—C3—C4—O4	13.8 (4)
O8—Mn1—O2—C2	−0.8 (4)	C1—C3—C4—O5	−167.5 (2)
O4—Mn1—O2—C2	−52.61 (17)	O1—C1—C5—C6	−54.3 (3)
O1—Mn1—O2—C2	27.93 (16)	C3—C1—C5—C6	−171.0 (2)
O9—Mn1—O4—C4	173.5 (2)	C2—C1—C5—C6	67.8 (3)
O6^i^—Mn1—O4—C4	9.9 (4)	Mn1^i^—O6—C6—O7	−5.4 (4)
O8—Mn1—O4—C4	−90.8 (2)	Mn1^i^—O6—C6—C5	171.83 (15)
O2—Mn1—O4—C4	78.9 (2)	C1—C5—C6—O7	−153.2 (2)
O1—Mn1—O4—C4	4.5 (2)	C1—C5—C6—O6	29.4 (3)
Mn1—O1—C1—C5	156.94 (15)	C15—N11—C11—C12	0.7 (4)
Mn1—O1—C1—C3	−85.35 (18)	N11—C11—C12—C13	−0.1 (4)
Mn1—O1—C1—C2	35.5 (2)	C15—C14—C13—C12	0.3 (4)
Mn1—O2—C2—O3	162.7 (2)	C15—C14—C13—C16	−177.6 (2)
Mn1—O2—C2—C1	−16.8 (3)	C11—C12—C13—C14	−0.4 (4)
O1—C1—C2—O3	165.9 (2)	C11—C12—C13—C16	177.4 (2)
C5—C1—C2—O3	43.6 (3)	C11—N11—C15—C14	−0.8 (4)
C3—C1—C2—O3	−76.0 (3)	C13—C14—C15—N11	0.3 (4)
O1—C1—C2—O2	−14.6 (3)	C14—C13—C16—C16^ii^	−174.0 (3)
C5—C1—C2—O2	−136.9 (2)	C12—C13—C16—C16^ii^	8.2 (5)

Symmetry codes: (i) −*x*+1, −*y*+1, −*z*+1; (ii) −*x*+2, −*y*+2, −*z*.

### Hydrogen-bond geometry (Å, º)

**Table:**

*D*—H···*A*	*D*—H	H···*A*	*D*···*A*	*D*—H···*A*
C12—H12···O3^iii^	0.95	2.48	3.344 (4)	151
C11—H11···O4^iv^	0.95	2.53	3.143 (3)	123
O9—H9*B*···O3^v^	0.93 (1)	2.56 (3)	3.113 (3)	118 (2)
O9—H9*B*···O2^v^	0.93 (1)	1.88 (1)	2.803 (3)	175 (3)
O9—H9*A*···O1*W*^vi^	0.93 (1)	1.78 (1)	2.695 (3)	170 (3)
O8—H8*B*···O5^vii^	0.93 (1)	1.78 (1)	2.707 (3)	173 (3)
O8—H8*A*···O7^vi^	0.93 (1)	1.87 (1)	2.769 (3)	163 (3)
O5—H5*O*···N11^viii^	0.86 (1)	1.76 (1)	2.625 (3)	178 (3)
O1*W*—H1*WB*···O3	0.93 (1)	1.92 (1)	2.843 (3)	175 (3)
O1—H1*O*···O6	0.86 (1)	1.88 (2)	2.616 (2)	143 (2)
O1*W*—H1*WA*···O7^ix^	0.93 (1)	2.08 (2)	2.891 (3)	145 (3)

Symmetry codes: (iii) *x*+1, *y*, *z*−1; (iv) *x*, *y*, *z*−1; (v) −*x*+1, −*y*+2, −*z*+1; (vi) *x*+1, *y*, *z*; (vii) −*x*+1, −*y*+1, −*z*+2; (viii) *x*, *y*, *z*+1; (ix) −*x*, −*y*+1, −*z*+1.
